# Comparison of digital and conventional impression techniques: evaluation of patients’ perception, treatment comfort, effectiveness and clinical outcomes

**DOI:** 10.1186/1472-6831-14-10

**Published:** 2014-01-30

**Authors:** Emir Yuzbasioglu, Hanefi Kurt, Rana Turunc, Halenur Bilir

**Affiliations:** 1 Department of Prosthodontics, School of Dentistry, Istanbul Medipol University, Istanbul, Turkey

**Keywords:** Digital impression, Clinical efficiency, Patient comfort, Patient preference

## Abstract

**Background:**

The purpose of this study was to compare two impression techniques from the perspective of patient preferences and treatment comfort.

**Methods:**

Twenty-four (12 male, 12 female) subjects who had no previous experience with either conventional or digital impression participated in this study. Conventional impressions of maxillary and mandibular dental arches were taken with a polyether impression material (Impregum, 3 M ESPE), and bite registrations were made with polysiloxane bite registration material (Futar D, Kettenbach). Two weeks later, digital impressions and bite scans were performed using an intra-oral scanner (CEREC Omnicam, Sirona). Immediately after the impressions were made, the subjects’ attitudes, preferences and perceptions towards impression techniques were evaluated using a standardized questionnaire. The perceived source of stress was evaluated using the State-Trait Anxiety Scale. Processing steps of the impression techniques (tray selection, working time etc.) were recorded in seconds. Statistical analyses were performed with the Wilcoxon Rank test, and p < 0.05 was considered significant.

**Results:**

There were significant differences among the groups (p < 0.05) in terms of total working time and processing steps. Patients stated that digital impressions were more comfortable than conventional techniques.

**Conclusions:**

Digital impressions resulted in a more time-efficient technique than conventional impressions. Patients preferred the digital impression technique rather than conventional techniques.

## Background

The introduction of computer-aided design/computer aided manufacturing (CAD/CAM) technology in dentistry has resulted in more accurate manufacturing of prosthetic frameworks, and greater accuracy of dental restorations, and the technology has improved since the 1980s [1,2]. The development strategy of CAD/CAM techniques included automating the production process and optimizing the quality of restorations by using new biocompatible materials, especially high performance ceramics, such as zirconia and lithium disilicate [3]. Several reports have demonstrated the potential for accurate and precise restorations using CAD/CAM technology [4-7].

According to the 8th edition of The Glossary of Prosthodontics Terms, “impression” is defined as “a negative likeness or copy in reverse of the surface of an object; an imprint of the teeth and adjacent structures for use in dentistry” [8]. The accuracy of the impression depends on the materials themselves [9-13], impression tray types [14-16], and impression techniques [17-19]. Each step in the process introduces potential human and/or material error [20,21].

There is some variability in impressions and the resulting master casts, depending on the technique and material used by the operator [22]. The accuracy of master casts has been the subject of numerous research projects, and is dependent on numerous items, including the water/powder ratio, vacuum versus hand mixing [23-25], and the type of dental stone and its compatibility with impression materials [26].

Digital impression and scanning systems were introduced in dentistry in the mid 1980s. It was predicted that most of the dentists in the U.S. and Europe would be using digital scanners for taking impressions within the next decade [27]. Digital impressions offer speed, efficiency, ability of storing captured information indefinitely and transferring digital images between the dental office and the laboratory [28]. The advantages of the digital impressions and scanning systems are improving patient acceptance, reducing the distortion of impression materials, 3D pre-visualization of tooth preparations, and potential cost- and time-effectiveness [29].

Several studies on the accuracy of intraoral scanners and digital impressions have been published, testing single-unit restorations [30-33], several teeth in a row [34-36], quadrants [37], and full arch scans [38,39].

A recent report by Lee & Gallucci [40] compared the operator’s preference of digital versus conventional implant impression techniques. In this in vitro study, inexperienced students made impressions on a customized model instead of live patients. The overall perception of the inexperienced students was that they preferred the digital impression technique. Until now there have been no clinical studies comparing the digital and conventional impression techniques.

The aim of this clinical trial was to evaluate the effectiveness, clinical outcomes, and patients’ preferences and attitudes towards the digital impression technique compared to the conventional impression technique. The first null hypothesis was that there is no difference in effectiveness and clinical outcomes between the conventional and digital impression techniques. The second null hypothesis was that there is no difference in patients’ preference and treatment comfort between the conventional and digital impression techniques.

## Methods

### Study design & patient selection

A controlled clinical trial was designed. The study population consisted of first year dental and medical students of the İstanbul Medipol University who had no experience with either conventional or digital impressions. The subjects were informed in detail about the possible risks and benefits, and all signed an informed consent form. The study was performed following the principles outlined in the Declaration of Helsinki on experimentation involving human subjects. The study protocol was reviewed and approved by the Ethical Committee of the Istanbul Medipol University, Istanbul, Turkey, (No:10840098-74).

### Inclusion/exclusion criteria

Twenty-four subjects (12 females, 12 males, aged 21.87 ± 2.76 years) who fulfilled the following inclusion criteria were recruited after an initial examination: no experience with either conventional or digital impressions, good general health, good oral hygiene, no periodontal disease, and good mental health. Prerequisites for exclusion in the study were previous impression experience, fixed or removable prosthetic rehabilitation, orthodontic treatment and preventive appliances, history of use of space maintainers in mixed dentition, moderate to excessive dental anxiety.

### Clinical scenario

A clinical scenario of an “excessive destruction of a mandibular molar and crown fracture of the lateral incisor, which would be restored by post-core and all ceramic crowns” was explained to the subjects during their orientation to the clinical settings of the study. Subjects watched an informational video illustrating the restorative steps of the clinical scenario. The impression phase was excluded from the video.

### Conventional impressions

One operator selected the proper tray for both arches of the subject, and applied the adhesive (Polyether Tray Adhesive, 3 M ESPE, Dental Products, St. Paul, MN, U.S.A.). The conventional impressions of mandibular and maxillary arches were made by polyether impression material (Impregum Penta Soft Quick Step MB, 3 M ESPE, Dental Products, St. Paul, MN, U.S.A.) with stock trays using the monophase impression technique. The interocclusal relationship was recorded with a polysiloxane bite registration material (Futar D, Kettenbach GmbH & Co. KG, Eschenburg, Germany). All materials were used according to the manufacturers’ guidelines and performed by the same operator (E.Y.).

**Figure 1:**
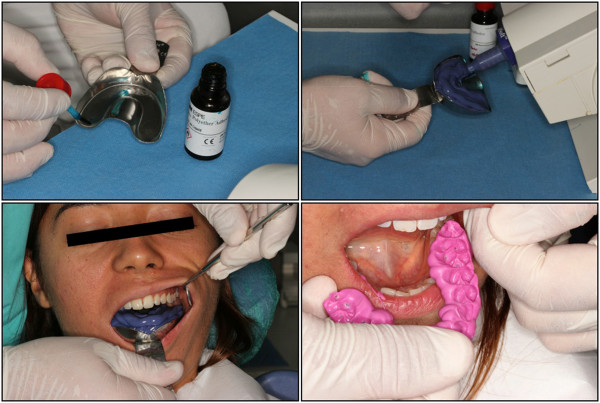
**Conventional impression technique.** Conventional impression technique. **A)** Adhesive application, **B)** Impression tray loading, **C)** Upper and lower arches impression, **D)** Bite registration.

The effectiveness and clinical outcomes of the conventional impression technique was evaluated by measuring the total treatment time, including the individual steps (Figure 1): A) tray selection, B) adhesive application, C) upper/lower impression, D) bite registration. The treatment time was measured in seconds and recorded for each step by a second operator (R.T. & H.B.). Immediately after the impressions were made, the attitudes and perceptions of the subjects towards the conventional impression technique were evaluated using a standardized questionnaire. The subjects’ perceived source of stress was also evaluated using the State-Trait Anxiety Scale immediately after the impression technique.

### Digital impressions

A digital impression appointment was scheduled for the same patients 2–3 weeks following the conventional impressions. The digital impressions were performed with the chairside dental CAD-CAM system (Cerec OMNICAM, Sirona Dental GmBH, Wals Bei Salzburg, Austria). The digital impression electronic data constituents of the virtual models for both arches and bite registration were recorded. All digital scanning procedures were carried out according to the manufacturer’s guidelines and performed by the same operator (EY).

**Figure 2:**
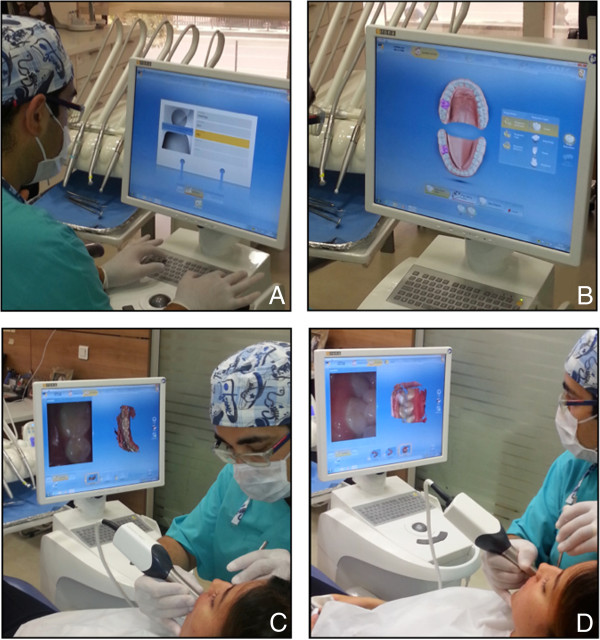
**Digital impression technique. A)** Entering patient information, **B)** Laboratory prescription, **C)** Upper and lower arches scanning, **D)** Bite scanning.

The effectiveness and clinical outcomes of the digital impression technique were evaluated by measuring the total treatment time, including the individual steps (Figure 2): A) entering patient information (including name, last name, date of birth, B) laboratory prescription (including shade of restoration, material choice of restoration, form of restoration), C) upper/lower scan, and D) bite scan. Treatment time was measured in seconds and recorded for each step by a second operator (R.T. & H.B.). Immediately after the impressions were made, the attitudes and perceptions of the subjects towards the digital impression technique were evaluated using a standardized questionnaire. The subjects’ perceived source of stress was also evaluated using the State-Trait Anxiety Scale immediately after the impression technique.

The subjects were also asked to answer a 9-item comparative questionnaire including the following research questions: Which was the preferred impression technique? Which was the recommended impression technique? Which impression technique was more efficient? Which impression technique would be most comfortable regarding impression techniques?

### Reliability and validity of questionnaires

The questionnaires used in this study were pre-tested, revised, and retested before use. A pilot questionnaire was tested on a representative sample of 10 patients. Test-retest reliability was performed to test the reliability and internal consistency of the questionnaires. The Cronbach Alpha reliability coefficient of the scales were found as 0.921, and 0.982, respectively. The adaptation, reliability and validity of the Turkish version of the State-Trait Anxiety Scale were evaluated by Öner and Le Compte in 1983 [41].

### Statistical analysis

Statistical analysis by the Wilcoxon Signed-Rank Test, with p = 0.05 as the level for statistical significance, was performed to evaluate the differences in effectiveness and clinical outcomes between conventional and digital impression techniques, using the SPSS 15.0 for Windows statistical software (SPSS Inc., Chicago, IL, USA).

The attitudes and perceptions of the subjects on both impression techniques were assessed with a self-administrated questionnaire using a Visual Analog Scale (VAS) ranging from 0 to 100. The data were analyzed statistical by the Wilcoxon Signed-Rank Test, with p = 0.05 as the level for statistical significance, using the SPSS 15.0 statistical software (SPSS Inc., Chicago, IL, USA).

The subjects’ preferences for the impression techniques were assessed with a 9-item comparative questionnaire, and the distribution of the answers were evaluated by descriptive analysis using the SPSS 15.0 statistical software (SPSS Inc., Chicago, IL, USA).

## Results

The evaluation of the effectiveness and clinical outcomes for both impression techniques are presented in Table 1. The mean overall treatment times were statistically significantly different (p < 0.001), and comparison of the mean impression times indicated a statistically significant difference (p < 0.001). The mean tray selection time for the conventional impression technique and the mean time for entering patient information for the digital impression technique were not statistically significant (p > 0.05). The mean adhesive application time for the conventional impression technique was statistically significantly different (p < 0.001) from the mean time for entering the laboratory prescription time for the digital impression technique. The difference between the mean bite registration time for the conventional technique and the mean bite scan time for the digital technique was statistically significant (p < 0.001).

**Table 1:** Scores of clinical efficiency outcomes of impression techniques

***Efficiency***	**Conventional**	**Digital**	**P-value**
Tray selection/Patient information	18,87 ± 2,42	19,08 ± 3,57	>0.05
Adhesive application/Laboratory prescription	27,75 ± 3,12	13,63 ± 1,98	<0.001*
Upper impression/Upper scan	240,70 ± 16,38	102,14 ± 17,77	<0.001*
Lower impression/Lower scan	226,10 ± 10,89	98,94 ± 10,56	<0.001*
Bite registration/Bite scan	91,96 ± 10,74	14,68 ± 3,82	<0.001*
Total treatment time	605,38 ± 23,66	248,48 ± 23,22	<0.001*

All data are presented as mean ± SD. Measured time is recorded as seconds. *Statistical significance level p-0.05.

### Outcomes of conventional impressions

The mean overall treatment time of the conventional impression technique was 605.38 ± 23.66 s. The mean treatment times of the individual steps of the conventional impression technique was as follows: Mean tray selection time, 18.87 ± 2.42 s; mean adhesive application time, 27.75 ± 3.12 s. The mean conventional impression time of the upper and lower jaws was 240.70 ± 16.38 s and the mean bite registration time was 91.96 ± 10.74 s.

### Outcomes of digital impressions

The mean overall treatment time of the digital impression technique was 248.48 ± 23.48 s. The mean treatment times of the individual steps of the digital impression technique were as follows: the mean time for entering patient information, 19.08 ± 3.57 s, and the mean time for entering the laboratory prescription time, 13.63 ± 1.98 s. The mean digital impression time for the upper and lower jaws was 98.94 ± 10.56 s and the mean bite scan time was 14.68 ± 3.82 s.

### Patients’ preferences and self concerns

The evaluation scores and the level of concerns of the subjects regarding the impression techniques are presented in Table 2. The mean scores of the subjects’ evaluation criteria regarding the two impression techniques were significantly different (p < 0.001). The subjects’ level of self concern were evaluated by scores of STATI-TX 1. The mean scores were not statistically significant (p > 0.05). All the subjects preferred the digital impression technique (p < 0.001), and patients’ preferences regarding the impression techniques, according to the 9-item comparative questionnaire, are listed in Table 3.

**Table 2:** Participants’ evaluation scores and level of self concerns about impression techniques

***Evaluation (VAS score)***	**Conventional**	**Digital**	**P-value**
Overall discomfort of impression	59,00 ± 37,72	90,04 ± 18,37	<0.001*
Overall time of impression	65,10 ± 41,55	90,28 ± 18,36	<0.001*
Smell/Voice	54,90 ± 39,04	86,52 ± 21,16	<0.001*
Taste/Heat	54,20 ± 28,06	88,16 ± 19,76	<0.001*
Queasiness	48,20 ± 44,53	91,80 ± 20,37	<0.001*
Discomfort during mouth was opened	44,40 ± 36,21	88,04 ± 19,86	<0.001*
Discomfort in TMJ	55,90 ± 43,31	88,68 ± 19,83	<0.001*
Breathing difficulty	59,90 ± 37,90	87,32 ± 21,02	<0.001*
Teeth and Periodontal sensivity	47,10 ± 43,21	85,36 ± 23,70	<0.001*
Total evaluation score	507,25 ± 277,34	827,50 ± 171,11	<0.001*
***Level of self concern***			
Score of STATI-TX 1	41,33 ± 3,84	43,29 ± 3,89	>0.05

All data are presented as mean ± SD. Visual Analog Scale (VAS). *Statistical significance level p-0.05.

**Table 3:** Participants’ preferences about impression techniques according to the 9-item questionnaire

***Preferences***	**Conventional**	**Digital**
Which impression technique do you prefer in case of one more time for impression procedure?	%0	%100
Which impression technique is more comfortable from point of comparison of two impression procedure?	%0	%100
Which impression technique do you suggest in case of a friends’ need for impression making?	%0	%100
Which impression technique do you prefer from point of time involved with impression procedure?	%0	%100
Which impression technique do you prefer from point of feeling taste/smell or voice/heat during impression procedure?	%0	%100
Which impression technique do you prefer from point of the size of the intraoral scanner/impression tray used in your mouth during impression procedure?	%0	%100
Which impression technique do you prefer from point of having tooth/gingival sensitivity during impression procedure?	%0	%100
Which impression technique do you prefer from point of having difficulty in breathing during impression procedure?	%0	%100
Which impression technique do you prefer from point of having gagging reflex during impression procedure?	%0	%100

## Discussion

In this clinical trial, according to the clinical scenario, the digital impression technique was more efficient than the conventional impression technique. Thus, the first null hypothesis was rejected. The subjects also preferred the digital impression technique rather than the conventional impression technique because of its comfort. Thus, the second null hypothesis was also rejected.

The study population was standardized and homogenized by including subjects who had no experience with conventional or digital impressions in their dental history. To investigate the clinical outcomes of the two impression techniques, homogenizing the study population is an acceptable clinical research method to optimize objectivity and minimize bias. This approach is important to avoid reporting the bias of patients who had previous experience with the dental impression procedure.

In this present study, we focused primarily on the efficiency of the two impression techniques and the preference of the patients under controlled clinical conditions. Future investigations should include the assessment of the accuracy of the impressions produced by experienced versus non-experienced operators, comparison of using scanning powders versus non-powder scanning, and comparison of full arch and partial impressions.

There are some limitations of this study. The study was designed as a comparative-controlled clinical trial, and the sequence of the evaluation of the two impression techniques was chosen for psychological reasons. There is a 2–3-week interval between the two evaluation appointments. This time period was deemed sufficient to erase from memory an event or a process. The evaluation process focuses on the outcomes of the impression techniques by means of total treatment time in seconds, and the study does not analyze any differences in precision of the two impression techniques.

Another limitation of the study was that only one operator performed the impression techniques to avoid the possible inter-operator error, such as the prolonged processing time taken by an inexperienced operator. The main purpose of the study was to focus on the patients’ perceptions and comfort in using different impression techniques. Evaluation by a second operator was not preferred because of main purpose of the study. Further investigations are planned to evaluate the perceptions of patients treated by different dental specialties and operator experience to the digital impression technique.

The last limitation of this study is that it ignored the time factors involved in the conventional impression technique, such as pouring and mounting the cast, trimming the dies, painting the die spacer, etc. By eliminating these steps, time for the traditional workflow would be reduced significantly. Furthermore, the digital impression technique and digital workflow are designed as the “digital working model” directly from the intraoral scan, without any additional factors. By virtually eliminating the intermediate processes, error accumulation in treatment and in the manufacturing cycle is no longer an issue.

The results of this study have revealed clinical evidence that the digital impression technique can be applied successfully for the impressions of restorative procedures based on clinical outcomes and the patients’ preferences. However, this study was performed in a clinical scenario that excluded the effect of actual treatment conditions, perceived dental anxiety and stress associated with treatment. This is an additional limitation of this study.

The major advantage of digital impressions is reducing the chair time. The mean total treatment time (p < 0.001) and the subjects’ evaluation scores (p < 0.001) regarding the impression techniques were significantly different (Tables 1 and 2). Improving the level of the patients’ comfort and treatment acceptance (p < 0.001) were other advantages of the digital impression techniques (Tables 1 and 2). Digital impressions tend to reduce repeat visits and retreatment, while increasing treatment effectiveness [42]. Patients will benefit from more comfort and a pleasant experience in the dentist’s chair.

The results of study indicate that the efficiency outcomes of the digital impression technique were higher than that of the conventional impression technique, with respect to treatment time taken up and the perceptions of the subjects. The effectiveness and clinical outcomes of both impression techniques (Table 1) were evaluated by recording the treatment time of each step in seconds, and were significantly different from each other (p < 0.001). The scores of the evaluation criteria regarding the two impression techniques (Table 2) that affect the subjects’ perception differed from one another in a statistically significant manner (p < 0.001).

The differences in the level of treatment comfort evaluated by the subjects, including breathing difficulty, queasiness, discomfort in the TMJ, and discomfort while the mouth was kept open were statistically significant (p < 0.001). Thus, the digital impression technique is more patient-friendly than the conventional impression technique. The results of this study present the major reasons why the subjects preferred the digital impression technique instead of the conventional impression technique (Table 3).

## Conclusions

Within the limitations of this study, the following conclusions can be drawn:

1. The digital impression technique was more efficient than the conventional impression technique. The overall treatment time for the conventional impression technique was longer than that for the digital impression technique. Thus, the first null hypothesis was rejected.

2. When compared with the conventional impression technique, the digital impression technique was accepted as the preferred and effective technique, according to the subjects’ perception. Thus, the second null hypothesis was rejected.

3. The treatment comfort of the digital impression technique was higher than that of the conventional impression technique when it was performed by an experienced operator.

## Competing interests

The authors declare that they have no competing interests.

## Authors’ contributions

EY is the designed and carried out the clinical study, collected the data for analysis , performed the statistical analysis and drafted the manuscript. HK participated in the design of the study and interpretation of data. RT and HB were collected the data for analysis. All authors read and approved the final manuscript.

## Pre-publication history

The pre-publication history for this paper can be accessed here:

http://www.biomedcentral.com/1472-6831/14/10/prepub
